# Habitat Differentiation and Trait Variation Across Disturbance Gradients in Coastal Plant Communities of the Andaman Coast, Thailand

**DOI:** 10.1002/ece3.73964

**Published:** 2026-07-03

**Authors:** Kanokporn Kaewsong, Ekaphan Kraichak, Chia‐Hao Chang‐Yang, Atiwat Boonrit, Prarop Plangngan, Sangsan Phumsathan

**Affiliations:** ^1^ Department of Conservation, Faculty of Forestry Kasetsart University Bangkok Thailand; ^2^ Department of Botany, Faculty of Science Kasetsart University Bangkok Thailand; ^3^ Department of Biological Sciences National Sun Yat‐sen University Kaohsiung Taiwan; ^4^ Department of National Parks, Wildlife and Plant Conservation Protected Areas Regional Office 5 Bangkok Thailand

**Keywords:** coastal ecosystems, environmental filtering, functional traits, peninsular Thailand, protected area management

## Abstract

Coastal sand dune ecosystems play a crucial role in ecological functions and biodiversity support. These ecosystems are shaped by complex environmental gradients and disturbance regimes that are difficult to quantify directly. However, tree communities in tropical Asian dunes remain poorly understood. Here, we examined tree community composition and functional traits as integrative responses to underlying drivers across 24 plots arranged along transects extending inland from the shoreline at Hat Thai Mueang Coastal Sand Dune, Thailand. We measured structural attributes, diversity metrics, and community‐weighted mean (CWM) functional traits and analyzed community composition using Bray–Curtis dissimilarity. Four distinct habitat types were identified, with distance from the sea emerging as the primary axis of community differentiation. Differences among habitats were consistent with inferred variation in hydrological regimes, particularly in an inland swamp subject to seasonal flooding. This inland swamp exhibited high wood density and low species diversity, suggesting strong environmental filtering under seasonally inundated conditions. In contrast, habitats outside the seasonally flooded swamp supported higher species diversity and lower wood density. Our findings indicate that coastal dune tree communities are structured by interacting disturbance regimes across environmental gradients, with wood density emerging as an important indicator of community assembly. These patterns can inform management strategies to maintain biodiversity and ecosystem resilience in protected coastal landscapes.

## Graphical abstract

Tree communities in a tropical coastal dune system exhibited distinct habitat differentiation across a sea‐to‐land disturbance gradient. These habitat differences were reflected in species composition, species diversity, and community‐weighted mean wood density.

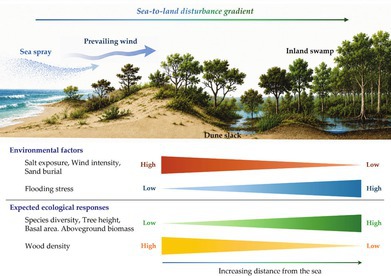

## 1 Introduction

Coastal sand dune ecosystems are shaped by recurrent physical disturbances and strong abiotic constraints associated with coastal dynamics, particularly sediment dynamics, wind exposure, and marine‐derived salinity (Bermúdez et al. 2024; Ciccarelli and Bona 2022; Hesp 2002; Houle 1997; La Bella et al. 2024; Rivera et al. 2025). These disturbance‐driven systems provide critical economic (Richardson and Nicholls 2021; Schlacher et al. 2007; Zhao et al. 2024) and ecological services, including shoreline stabilization, sediment retention, and habitat support for diverse flora and fauna (Bermúdez et al. 2024; Hesp 2002; Houle 1997; La Bella et al. 2024). However, repeated physical disturbance creates highly dynamic environments in which tree communities are required to persist (Ding et al. 2025; Nanko et al. 2019; Turner et al. 2012). As a result, coastal dune ecosystems are particularly sensitive to external pressures such as climate change and recreational use (Richardson and Nicholls 2021; Zhao et al. 2024). Understanding how tree communities organize along disturbance gradients is therefore essential for informing conservation and restoration strategies (Chollet et al. 2023; De Battisti 2021), particularly across pronounced sea–inland environmental gradients.

Along coastal sand dunes, environmental gradients arise from interacting disturbance processes that vary with distance from the shoreline. Nearshore habitats are typically exposed to greater wind intensity, salt spray deposition, and sediment redistribution, which together constrain plant establishment, growth, and survival (Bermúdez et al. 2024; Ciccarelli and Bona 2022; Hesp 2002; Houle 1997; La Bella et al. 2024). Sand movement and burial affect rooting depth and seedling establishment (La Bella et al. 2024), salt spray alters plant water relations and imposes physiological constraints (Du and Hesp 2020; Munns and Tester 2008), and wind exposure influences plant architecture and mechanical stability (Hesp 1991). These interacting processes generate distinct microhabitats along the shoreline–inland gradient and drive shifts in species composition and structural traits (Chollet et al. 2023; Houle 1997). In addition to aboveground processes, coastal dune systems are also influenced by water table dynamics that generate temporary flooding or waterlogging in dune slacks. Such hydrological variability, driven by fluctuations in the water table and episodic rainfall, represents an additional environmental filter that can strongly influence vegetation dynamics (Dwyer et al. 2021; Hernández‐Cordero et al. 2022; Schat and van Beckhoven 1991; Zunzunegui et al. 2022). In this study, the term disturbance gradient refers to the spatial variation in naturally occurring coastal stressors, including salt spray, wind exposure, sand movement, and hydrological influences. These factors are expected to vary across the coastal dune landscape and may contribute to differences in species establishment, persistence, and community composition.

Trait‐based approaches provide insight into how tree communities respond to environmental filters operating in disturbance‐prone landscapes (Kichenin et al. 2013; Kraft et al. 2008; Lasky et al. 2013; McGill et al. 2006; Swenson et al. 2012; Violle et al. 2007; Xu et al. 2017). Functional traits associated with mechanical resistance and growth strategies, particularly wood density, reflect species' capacity to persist under repeated physical disturbance and maintain structural stability (Chave et al. 2009; Cornelissen et al. 2003; Kraft et al. 2008; Pérez‐Harguindeguy et al. 2013; Wright et al. 2010). Variation in wood density across dune habitats can therefore reveal mechanisms of tree community assembly along disturbance gradients, potentially favoring species with higher wood density in disturbance‐exposed shoreline habitats. Variation in plant stature may further modify airflow and sand movement (Jacobs et al. 1995; Niedoroda et al. 1991), generating fine‐scale structural heterogeneity that promotes spatial clustering of species (Hesp 2002; Kim et al. 2014). Such spatial clustering can, in turn, influence local species diversity by shaping patterns of dominance and coexistence within stands. In highly disturbed microsites, clustering of species with similar stress‐tolerant traits may reduce local diversity through competitive dominance, whereas in less exposed or structurally heterogeneous areas, clustering may facilitate niche partitioning and promote species coexistence. By shaping stand structure, trait‐driven spatial patterning may further influence aboveground biomass (Abanda et al. 2011; De Battisti 2021; Gu et al. 2025), consistent with evidence linking diversity and structural complexity to biomass accumulation via resource‐use complementarity and stand structural mediation (Wen et al. 2022).

Many studies on sand dune plant communities have focused on grass‐ or herb‐dominated systems (Bermúdez et al. 2024; Ciccarelli and Bona 2022; Dwyer et al. 2021; Jay et al. 2025; La Bella et al. 2024; Oosting 1945; Pickart 2021; Valcheva et al. 2021), with several examining functional traits (Bermúdez et al. 2024; Ciccarelli and Bona 2022; La Bella et al. 2024). In contrast, tree‐dominated dune systems remain poorly understood, particularly in tropical Asian coastal landscapes. This gap limits our ability to predict how tree communities assemble and persist under disturbance, constraining trait‐based inference and spatially explicit management of dune ecosystems. To address this gap, our study focuses on the Hat Thai Mueang coastal sand dune within Khao Lampi–Hat Thai Mueang National Park, a protected area along Thailand's Andaman coast characterized by high biodiversity and one of the country's remaining intact coastal forests. The national park forms part of the Andaman Sea Nature Reserves of Thailand, currently included on Thailand's UNESCO World Heritage Tentative List, and supports important coastal habitats such as nesting beaches for sea turtles (Department of National Parks, Wildlife and Plant Conservation 2022). In this study site, we examine how species composition, structural characteristics, and community‐weighted mean (CWM) functional traits vary along sea–inland environmental gradients. By integrating community composition analysis, habitat classification, and trait‐based approaches, we aim to identify the environmental filters and spatial factors that influence the structure of tree communities in coastal dune ecosystems.

**FIGURE 1:**
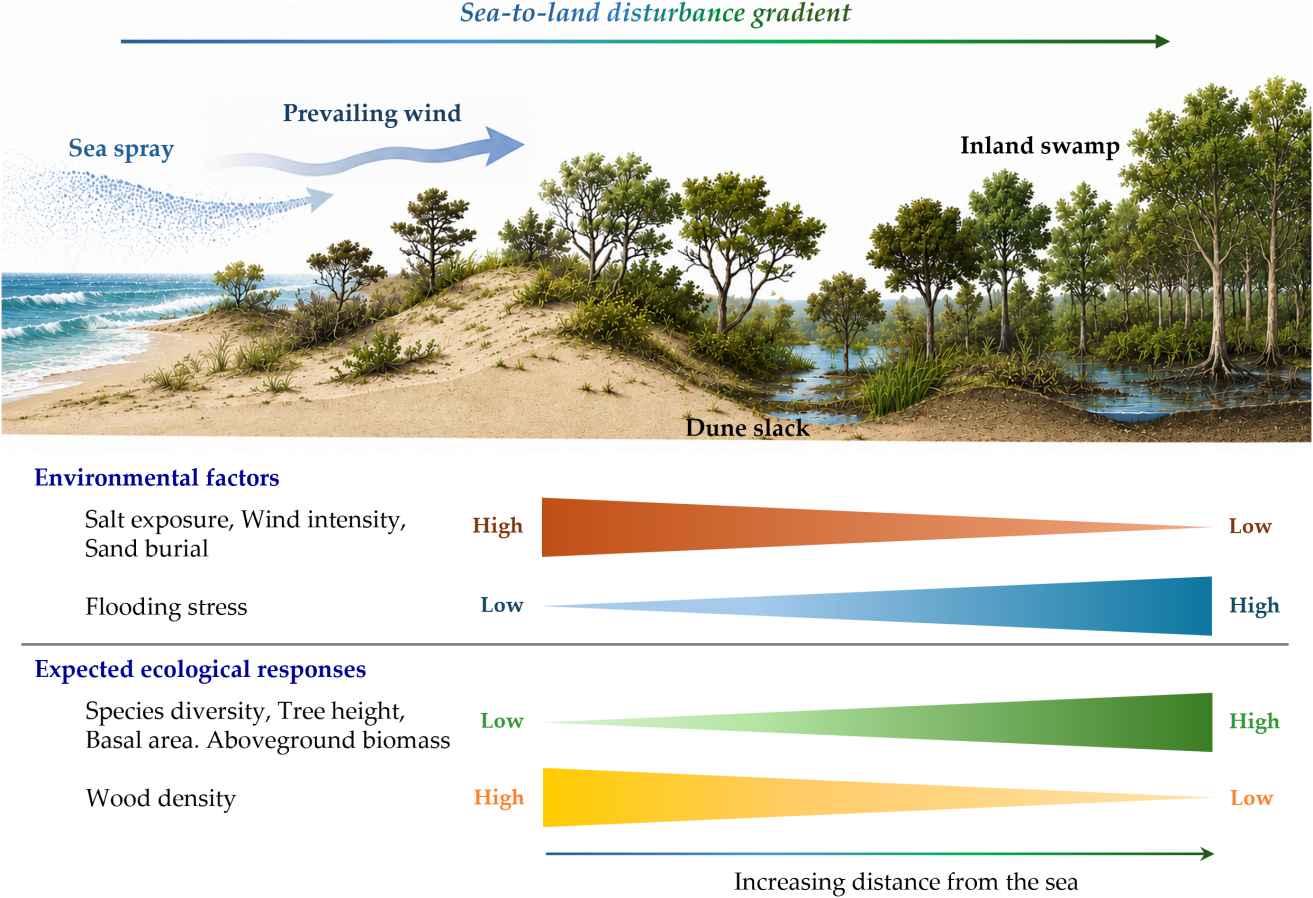
Conceptual representation of the sea‐to‐land disturbance gradient in the Hat Thai Mueang coastal dune system. Salt spray, wind exposure, sand movement, and flooding stress are hypothesized to vary along the shoreline–inland gradient, generating distinct environmental conditions from shoreline habitats to inland swamp environments. These processes may influence tree community composition, diversity, structural characteristics, and functional traits.

We hypothesize that tree communities in dune habitats experiencing different disturbance regimes, shaped by recurrent physical disturbance, exhibit structural and functional trait compositions that enhance persistence under these conditions. Specifically, we expect distinct habitat types to emerge along sea–inland gradients, with habitats experiencing contrasting disturbance regimes tending to differ in species diversity and functional traits, particularly wood density (Figure 1). To investigate these patterns, four questions guide this study: (1) what distinct habitat types emerge based on species composition; (2) to what extent environmental gradients, disturbance processes, and spatial position explain variation in community composition; (3) how do species diversity and structural metrics (basal area and aboveground biomass) vary among habitat types; and (4) how do community‐level functional traits (CWM wood density and CWM maximum height) differ across habitats experiencing different disturbance regimes.

## 2 Methods

### 2.1 Overview of the Study Area

The study area is located in Khao Lampi‐Hat Thai Mueang National Park, in peninsular Thailand. The national park, covering 72 km^2^, is recognized under the IUCN protected area category system and is divided by Phetkasem Road into two zones: (1) the coastal zone (about 15 km^2^), which includes 13.6 km‐long beach with coastal plant communities and one of the country's most intact coastal forests; and (2) the upland forest zone (about 57 km^2^), characterized by tropical rainforest. The coastline runs obliquely from northwest to southeast rather than strictly north–south, and the coastal zone varies in width from approximately 350 to 1600 m. This area also serves as an important nesting site for four marine turtle species, including the leatherback turtle ( *Dermochelys coriacea* ). At the northern end, the beach terminates in a prominent headland, while a large brackish canal along the eastern boundary supports mangrove vegetation. Parts of the central coastal zone experience seasonal inundation during the rainy season, with water retained for short periods following intense rainfall. This episodic hydrological disturbance is associated with the dominance of *Melaleuca cajuputi* in low‐lying areas.

Based on long‐term climate records, the study area experiences a strong monsoon climate, with a mean annual rainfall of 3657 mm. A short dry period occurs from December to February, during which monthly rainfall averages less than 100 mm. The heaviest rainfall typically occurs between August and October, with a mean annual temperature of 27.4°C. Soils in the coastal zone are predominantly coarse‐textured and sandy. The area was partially affected by the Indian Ocean earthquake and tsunami in 2004 (Department of National Parks, Wildlife and Plant Conservation 2022).

### 2.2 Sample Plot Design and Tree Data

**FIGURE 2:**
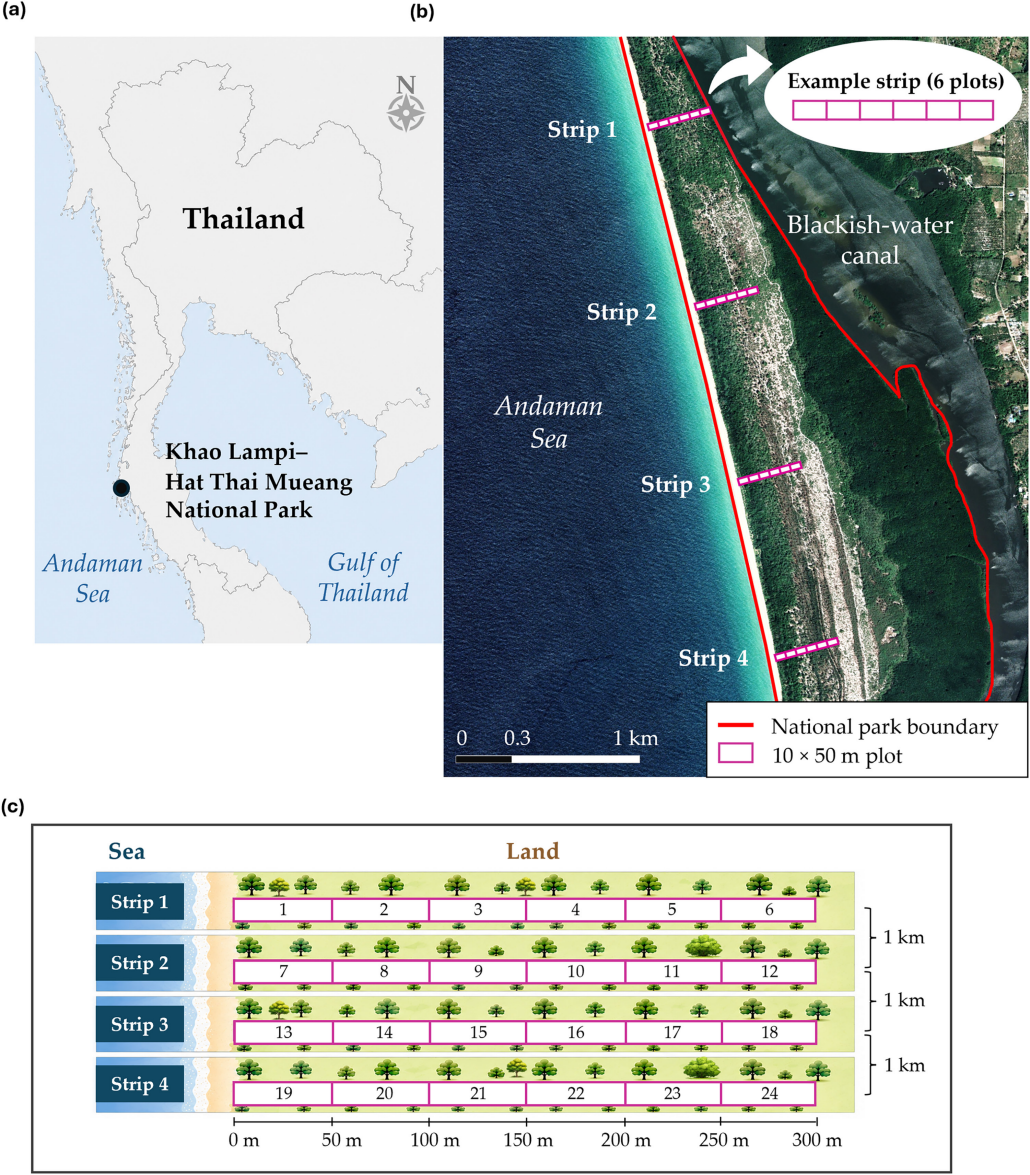
Study area and sampling design in Khao Lampi‐Hat Thai Mueang National Park, Thailand: (a) location of the national park in southern Thailand; (b) coastal study area showing the four sampling strips; (c) schematic representation of the sampling design, consisting of four strips spaced 1 km apart, each containing six 10 × 50 m plots positioned at 50 m intervals along a gradient perpendicular to the shoreline.

Tree community data were obtained from systematically arranged sample plots arranged along a sea‐to‐land gradient within the coastal zone of Khao Lampi–Hat Thai Mueang National Park between 2015 and 2017 (Boonrit 2020). The study area covers 1.2 ha and is located at 8°30′7.26′′ N, 98°13′22.3′′ E. The sampling design consisted of four 10 × 300 m transects spaced 1 km apart along the coastline. Each transect was subdivided into six 10 × 50 m plots positioned at 50 m intervals inland along a gradient perpendicular to the shoreline (Figure 2).

Distance from the sea was measured perpendicular to the coastline to accurately represent shoreline proximity. GPS coordinates were recorded for all 24 plots using a handheld Garmin GPS receiver, with positional accuracy typically within ±3–5 m under suitable satellite reception conditions. Within each plot, all trees with diameter at breast height (DBH) ≥ 4.7 cm were tagged, measured for DBH and total height, and identified to species level. Tree height was measured using a laser rangefinder and recorded in meters. The DBH threshold of 4.7 cm followed the original sampling protocol, in which individuals ≥ 4.7 cm DBH were classified as trees and measured in detail, whereas seedling and sapling stages were surveyed using different protocols and did not include the full set of variables required for the present analyses. The final dataset comprised 1257 individuals representing 49 species (Tables S1 and S2). Species names were standardized, and data were screened for measurement inconsistencies prior to analysis. Elevation was recorded for each plot using GPS and included as an environmental variable to account for potential microtopographic effects on species distribution.

### 2.3 Structural Metrics and Functional Trait Composition

Structural metrics and functional traits of tree communities were quantified using basal area, aboveground biomass, wood density, and maximum height.

Basal area was calculated for each individual tree using the formula:

(1) $$\text{Basal area}=\pi \times {\left(\mathrm{DBH}\right)}^{2}/40,000$$

where DBH is in cm, resulting in basal area expressed in m^2^.

Aboveground biomass was estimated using the allometric equation from Chave et al. (2014):

(2) $$\text{Aboveground biomass}=0.0673\times {\left(\rho \times \mathrm{DBH}\times H\right)}^{0.976}$$

where ρ is wood density (g/cm^3^), *H* is total height (m), and DBH is in cm.

Wood density values were obtained from the Global Wood Density Database (Zanne et al. 2009). Species‐level data were used when available. If species‐level data were not available, wood density was estimated using genus‐level means. If genus‐level means were also unavailable, family‐level means were applied. The proportion of species assigned at each taxonomic level was 53% at the species level, 41% at the genus level, and 6% at the family level.

Maximum height for each species was determined as the tallest recorded individuals of that species within the dataset. Values were expressed in meters.

To quantify functional trait composition at the community level, CWM wood density and CWM maximum height were calculated for each plot using species' basal area as the weighting factor (Garnier et al. 2004; Lavorel et al. 2008):

(3) $$\mathrm{CWM}=\sum_{i=1}^{n}\left(\mathrm{pᵢ}\times \mathrm{tᵢ}\right)$$

where *p*_*i*_ is the proportion of total basal area in the plot contributed by species *i*, *t*_*i*_ is the trait value for species *i*, *n* is the total number of species, and CWM represents the plot‐level community‐weighted mean trait value.

### 2.4 Community Composition Analysis and Habitat Classification

Tree community dissimilarity among plots was calculated using Bray‐Curtis distance on the summed basal area of each species per plot. Basal area was used instead of individual counts to better represent forest structure and biomass contribution. The resulting dissimilarity matrix was then ordinated using metric multidimensional scaling (classical MDS) implemented with the cmdscale function, after calculating distances with the vegdist function in the “vegan” package in R (Oksanen et al. 2015). To classify plots into distinct habitat types, k‐means clustering was applied to the ordination scores. Using the reduced two‐dimensional ordination space allowed clustering based on the major gradients of species composition while minimizing noise and high‐dimensional complexity inherent in the full species matrix. The optimal number of clusters was evaluated using average silhouette width across a range of *k* values (*k* = 2 to 10). When alternative solutions showed similar support, ecological interpretability was also considered in selecting the final clustering solution. The final k‐means clustering was performed with *k* = 4, using a random seed of 123 and 25 random initializations (nstart = 25) to improve clustering stability. Each plot was assigned to one of the resulting clusters, which were treated as distinct habitat types.

We further enhanced the interpretation of beta diversity patterns by constructing convex hulls around each cluster in the MDS ordination space. These polygons visually delineated the spatial extent and separation of community types, providing intuitive insight into their compositional distinctiveness, including potential overlaps or separation among habitat types. Visualization was performed using the “ggplot2” package in R (Wickham 2016). To support the visual interpretation from the convex hull plots, differences in community composition among clusters were tested using permutational multivariate analysis of variance (PERMANOVA) (Anderson 2001) based on a Bray–Curtis dissimilarity calculated from a species‐by‐plot basal area matrix. Statistical significance was assessed using 999 permutations implemented in the adonis2 function of the vegan package. Multivariate differences in dispersion among habitat types were assessed using analysis of multivariate homogeneity of group dispersions (PERMDISP) (Anderson 2006), implemented using the betadisper function in the vegan package. Statistical significance was evaluated using 999 permutations via the permutest function. Pairwise differences among groups were further examined using Tukey's Honest Significant Difference (HSD) test. Analyses were conducted using the “vegan” package in R (Oksanen et al. 2015).

### 2.5 Environmental Disturbance and Spatial Analysis

We assessed the influence of environmental and spatial structure on tree community composition using Mantel tests (Mantel 1967) based on Bray–Curtis dissimilarity derived from the summed basal area of all trees per plot. Environmental distances were computed as Euclidean distances between plots for two variables: (1) distance from the shoreline, used as a proxy for a sea–inland gradient integrating multiple disturbance‐related environmental processes; and (2) elevation, which may influence microhabitat conditions and local hydrological disturbance. Flooding regimes, groundwater influence, and soil conditions were not quantified directly and are discussed only as inferred environmental factors based on field observations and previous descriptions of the study area. Spatial distances were calculated as Euclidean distances between plot coordinates (*x*, *y* in meters). Mantel tests were performed using Pearson correlation with 999 permutations to evaluate the relationship between community dissimilarity and environmental and spatial distance matrices. Analyses were conducted using the mantel function in the “vegan” package (Oksanen et al. 2015).

To further examine the combined effects of environmental and spatial factors, we conducted distance‐based redundancy analysis (db‐RDA). This multivariate approach allows simultaneous testing of multiple explanatory variables while accounting for the dissimilarity structure of community data. Distance from the shoreline, elevation, and spatial coordinates (UTMx and UTMy) were included as explanatory variables. UTM coordinates were used in their original units (meters). Significance was assessed using permutation‐based ANOVA with 999 permutations. Adjusted coefficients of determination (adjusted *R* ^2^) were calculated to estimate the proportion of variation explained by the model. Analyzes were performed using the capscale function in the “vegan” package (Oksanen et al. 2015).

### 2.6 Species Diversity Analysis

Species diversity variation among habitat types derived from k‐means clustering was assessed. Four diversity indices were calculated for each plot: (1) Shannon‐Wiener diversity index; (2) Simpson diversity index; (3) species richness; (4) Pielou's evenness index. For Shannon‐Wiener, Simpson, and Pielou's evenness indices, the basal area of all individuals belonging to the same species within a plot was summed, and species proportions were calculated based on each species' contribution to total plot basal area rather than stem abundance.

Shannon‐Wiener diversity index (*H*′):

(4) $$H^{\prime }=-\sum_{i=1}^{S}\left(\mathrm{pᵢ}\times \ln\left(\mathrm{pᵢ}\right)\right)$$

Simpson diversity index (*D*):

(5) $$D=1-\sum_{i=1}^{S}{\mathrm{pᵢ}}^{2}$$

Species richness index (*S*):

(6) S=Total number of species in the plot

Pielou's evenness index (*J*):

(7) $$J=H^{\prime }/\ln\left(S\right)$$

where *p*_*i*_ is the proportion of total basal area contributed by species *i* in the plot, *S* is the total number of species in the plot, *H′* is the Shannon‐Wiener diversity index, *D* is the Simpson diversity index, *J* is Pielou's evenness index. All indices were computed using functions in the “vegan” package (Oksanen et al. 2015).

Statistical differences among habitat types were tested using one‐way ANOVA for Shannon‐Wiener diversity index, Simpson diversity index, and Pielou's evenness index. Multiple pairwise comparisons were performed using Tukey's HSD post hoc test. Species richness violated the assumption of normality. Therefore, a Kruskal‐Wallis nonparametric ANOVA was applied, followed by Dunn's test with Benjamini‐Hochberg correction for multiple comparisons (Dinno 2015).

### 2.7 Statistical Analysis of Species Diversity, Structural Metrics, and Functional Trait Composition Among Habitat Types

To examine differences in plant community characteristics across the four habitat types identified by k‐means clustering, we analyzed species diversity indices, structural metrics, and CWM trait values. Statistical comparisons were conducted using either a one‐way ANOVA or a Kruskal‐Wallis test, depending on whether the assumptions of normality and homogeneity of variance were met.

#### 2.7.1 Species Diversity

Shannon‐Wiener, Simpson, and Pielou's evenness indices met parametric assumptions and were analyzed using one‐way ANOVA followed by Tukey's HSD post hoc test. Species richness did not meet these assumptions and was analyzed using a Kruskal–Wallis test with Dunn's post hoc comparisons and Bonferroni‐adjusted *p*‐values.

#### 2.7.2 Structural Metrics and Functional Trait Composition

Among all variables, only CWM wood density met the assumptions of ANOVA and was analyzed using one‐way ANOVA followed by Tukey's post hoc test. Basal area, aboveground biomass, and CWM maximum height violated the assumptions and were analyzed using the Kruskal‐Wallis test, followed by Dunn's post hoc comparisons with Bonferroni‐adjusted *p*‐values. Statistically significant groupings were visualized using the “ggplot2” package and grouping letters in the boxplots were obtained using the “multcompView” package. The “FSA” package was used for Dunn's post hoc comparisons. All analyses were conducted in R software (version 4.5.1) (R Core Team 2025).

## 3 Results

### 3.1 Identification of Habitat Types Based on Species Composition and Structure

**FIGURE 3:**
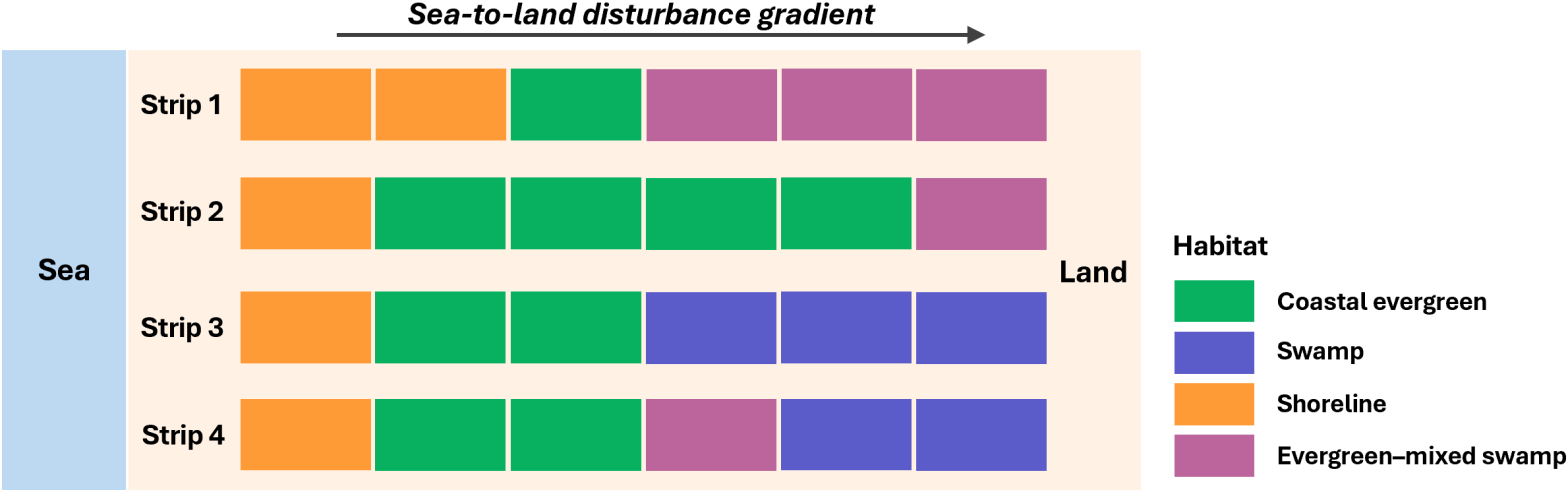
Habitat classification identified by k‐means clustering on classical MDS ordination of Bray–Curtis dissimilarity across four strips in Khao Lampi‐Hat Thai Mueang National Park. Colors indicate the four habitat types. Each rectangle represents a 10 × 50 m plot within each strip, illustrating the spatial arrangement of plots along the sea‐to‐land gradient.

**FIGURE 4:**
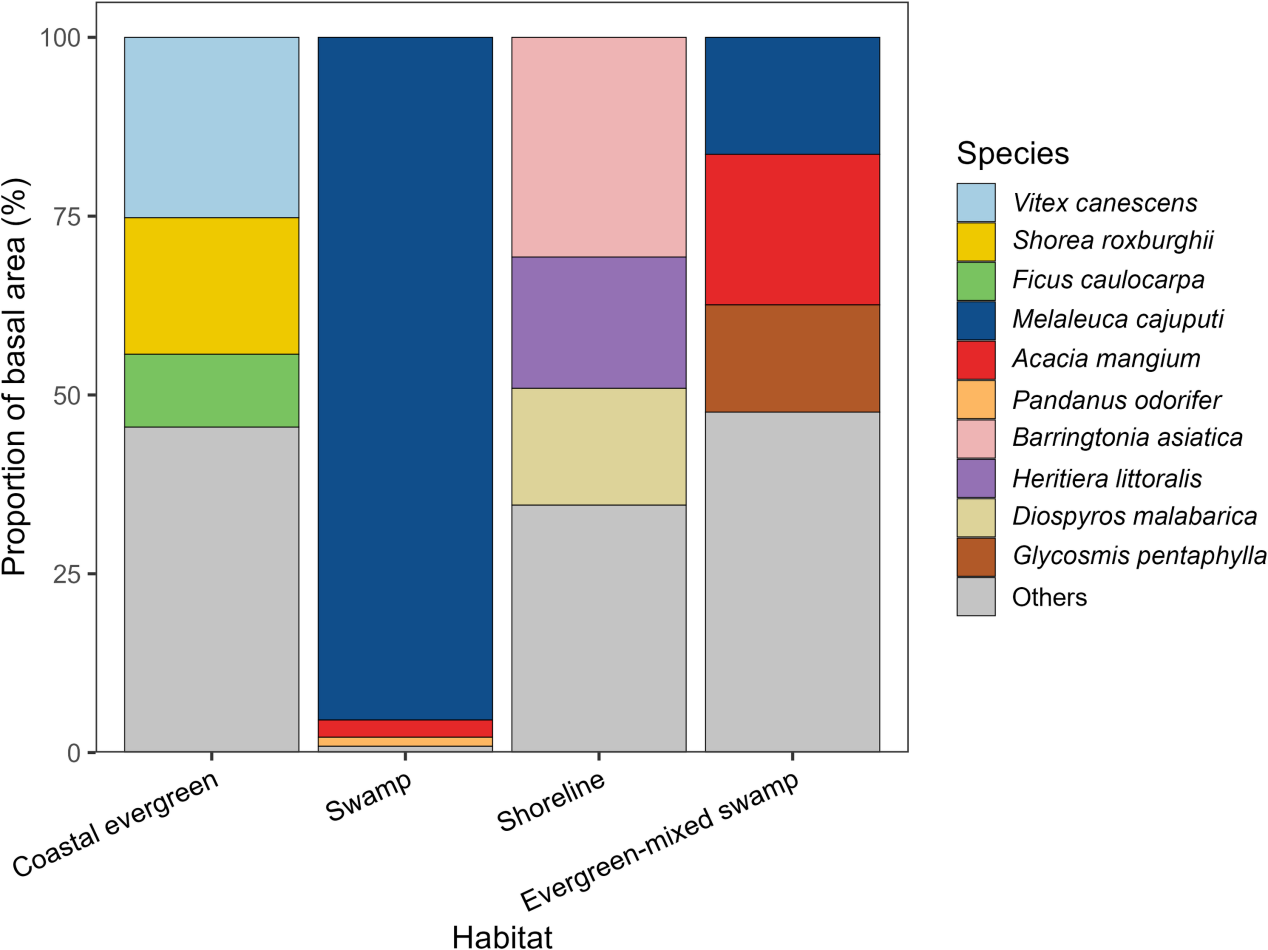
Stacked bar‐graph showing the proportions of the top three tree species in each habitat (based on basal area), with all remaining species grouped as “Others”, in Khao Lampi‐Hat Thai Mueang National Park, Thailand. Species composition varied among habitat types, with some species occurring across more than one habitat.

The first two ordination axes explained approximately 42% of the total variation in community composition. Based on clustering of the ordination scores, the plots were classified into four distinct habitat types (Figure 3). The resulting classification comprised nine plots in the coastal evergreen habitat and five plots each in the swamp, shoreline, and evergreen–mixed swamp habitats. Silhouette analysis yielded the highest mean silhouette width at *k* = 5 (0.613), followed closely by *k* = 4 (0.599). Given the small difference between solutions, *k* = 4 was retained because it provided greater ecological interpretability and clearer separation of community types. These habitat types differed in species composition, as evidenced by the relative basal area contributions of dominant tree species (Figure 4).

**FIGURE 5:**
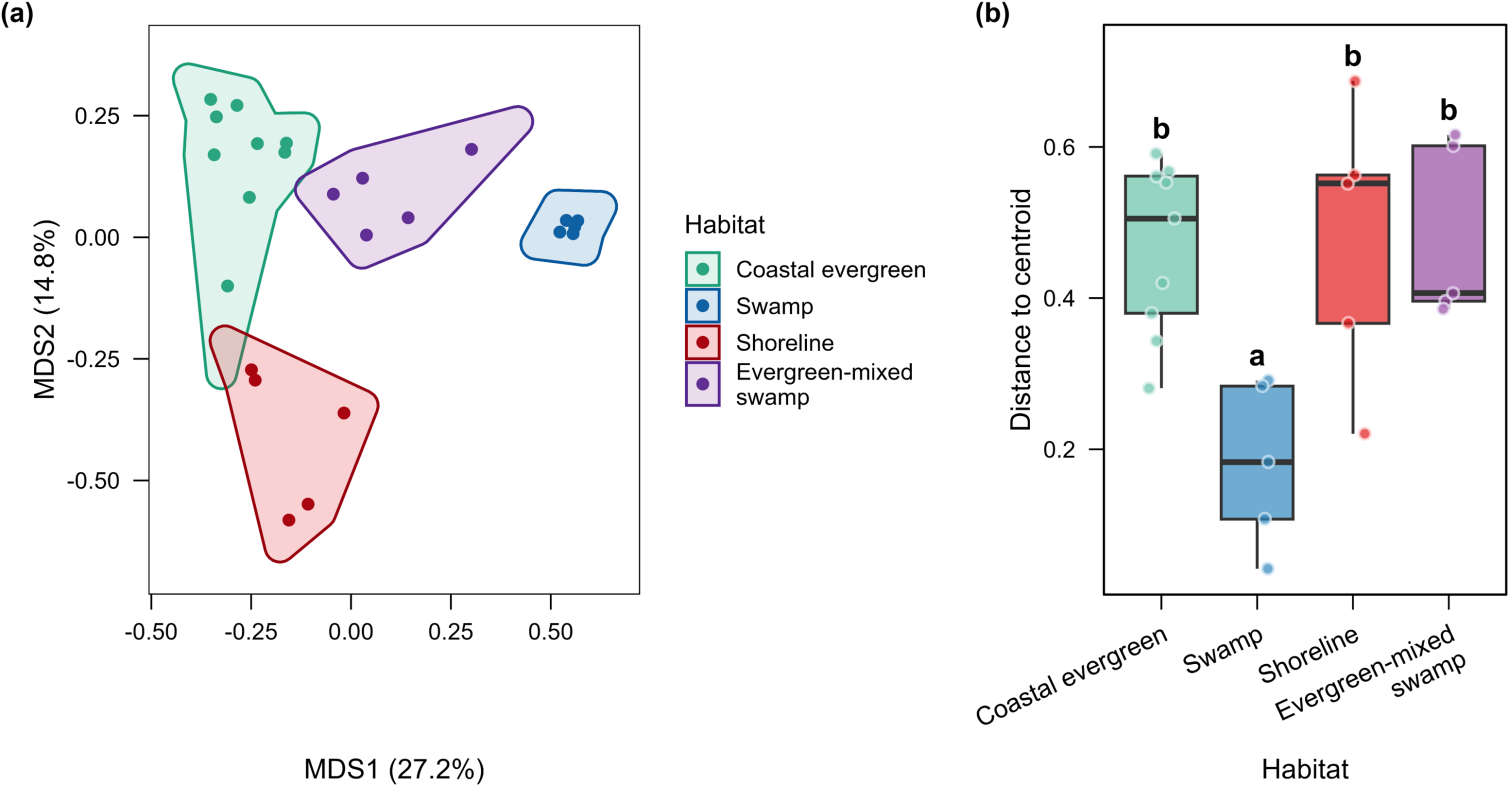
Tree community composition across four habitat types: (a) MDS ordination of plots grouped by k‐means clustering, with convex hulls delineating the compositional boundaries of each habitat; (b) boxplot of distances to centroid per habitat from PERMDISP analysis. The line within each box represents the median. The box edges represent the first and third quartiles. Whiskers extend to 1.5 times the interquartile range. Points show individual plot values, jittered for clarity. Sample sizes were coastal evergreen (*n* = 9), swamp (*n* = 5), shoreline (*n* = 5), and evergreen–mixed swamp (*n* = 5). Different letters above the boxes indicate statistically significant differences among habitats (Tukey's HSD, *p* < 0.05).

Consistent with these compositional patterns, the convex hull plot demonstrated clear spatial separation among habitat types (Figure 5a). The coastal evergreen habitat was located adjacent to the shoreline habitat, with a small area of overlap, indicating partial compositional similarity between these two habitats. The evergreen–mixed swamp habitat exhibited compositional characteristics similar to those of the coastal evergreen and shoreline habitats but did not overlap with any other habitat type. In contrast, the swamp habitat was the most spatially isolated, forming a tightly clustered group distinct from the other habitats. Multivariate dispersion differed significantly among the four habitat types, as revealed by PERMDISP (*p* = 0.003; Figure 5b). The swamp habitat exhibited the lowest average distance to centroid, indicating the lowest within‐habitat variability in community composition. In contrast, the coastal evergreen, shoreline, and evergreen–mixed swamp habitats showed higher average distances to centroid, reflecting greater within‐group heterogeneity. Pairwise comparisons using Tukey's HSD test showed that the swamp habitat was significantly less dispersed than the other habitat types. Consistent with these dispersion patterns, PERMANOVA confirmed significant differences in community composition among the four habitats (*R* ^2^ = 0.485, *p* < 0.001). Together, these results support the distinct community structures observed in the MDS ordination space and the convex hull plots, highlighting both differences in composition and within‐group variability among habitats.

### 3.2 Environmental Disturbance Stress and Spatial Driver

The mantel test revealed a significant correlation between Bray–Curtis dissimilarity and distance from the sea (*r* = 0.48, *p* = 0.001), indicating that species composition varied with proximity to the coastline. Bray–Curtis dissimilarity also showed a weak but statistically significant correlation with elevation (*r* = 0.18, *p* = 0.01), suggesting that microtopographic variation may influence tree community composition. A weak but significant correlation was also observed with spatial distance (*r* = 0.19, *p* = 0.01). This result implied weak spatial structuring in the tree community across the study area.

Based on the db‐RDA, distance from the sea, elevation, and x‐coordinate significantly influenced community composition and together explained 24% of the variation in community composition (adjusted *R* ^2^ = 0.24, *p* = 0.001), while the y‐coordinate was not significant. This suggested that environmental gradients and spatial position along the east–west axis contribute to variation in tree community structure (see Figure S1).

### 3.3 Variation in Species Diversity Among Habitat Types

**FIGURE 6:**
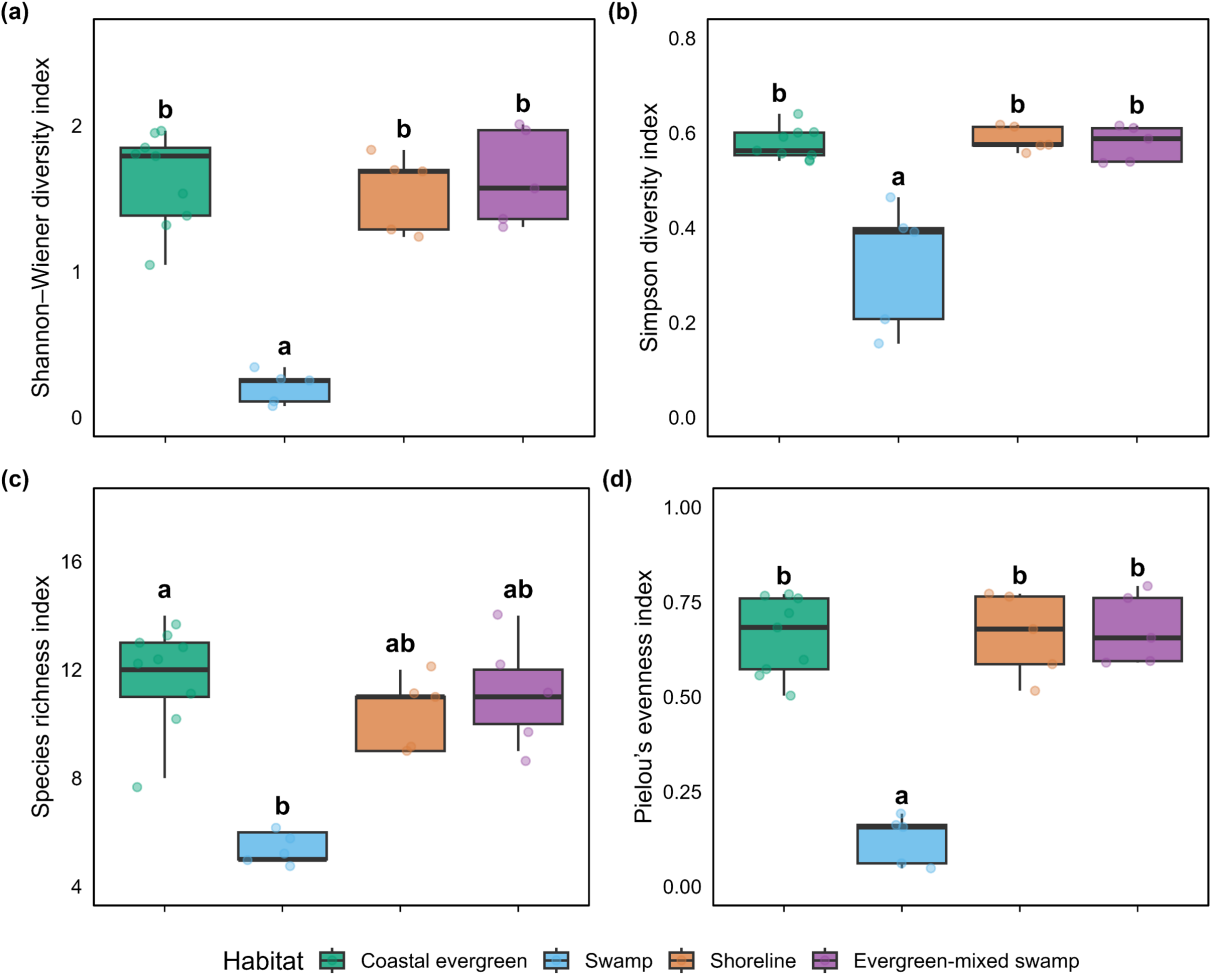
Boxplots comparing species diversity indices among habitat types: (a) Shannon‐Wiener diversity index; (b) Simpson diversity index; (c) Richness index; (d) Pielou's evenness index. The line within each box represents the median. The box edges represent the first and third quartiles. Whiskers extend to 1.5 times the interquartile range. Points show individual plot values, jittered for clarity. Sample sizes were coastal evergreen (*n* = 9), swamp (*n* = 5), shoreline (*n* = 5), and evergreen–mixed swamp (*n* = 5). Different letters above the boxes indicate statistically significant differences among habitats (Tukey's HSD or Dunn's test, *p* < 0.05, depending on the test used).

Species diversity differed significantly among the four habitat types (Shannon: *F* (3, 20) = 32.50, *p* < 0.001, η ^2^ = 0.83; Simpson: *F* (3, 20) = 19.44, *p* < 0.001, η ^2^ = 0.74; Richness: *χ* ^2^ (3) = 13.06, *p* = 0.005, η ^2^ *H* = 0.50; Evenness: *F* (3, 20) = 41.55, *p* < 0.001, η ^2^ = 0.86). The swamp habitat exhibited significantly lower Shannon diversity, Simpson diversity, and Pielou's evenness than the other habitat types (Figure 6). Overall, these results indicate that the swamp habitat supports less diverse and more uneven communities, whereas the coastal evergreen, shoreline, and evergreen–mixed swamp habitats generally exhibited higher diversity and evenness.

### 3.4 Variation in Functional Traits and Structural Characteristics Across Habitat Types

**FIGURE 7:**
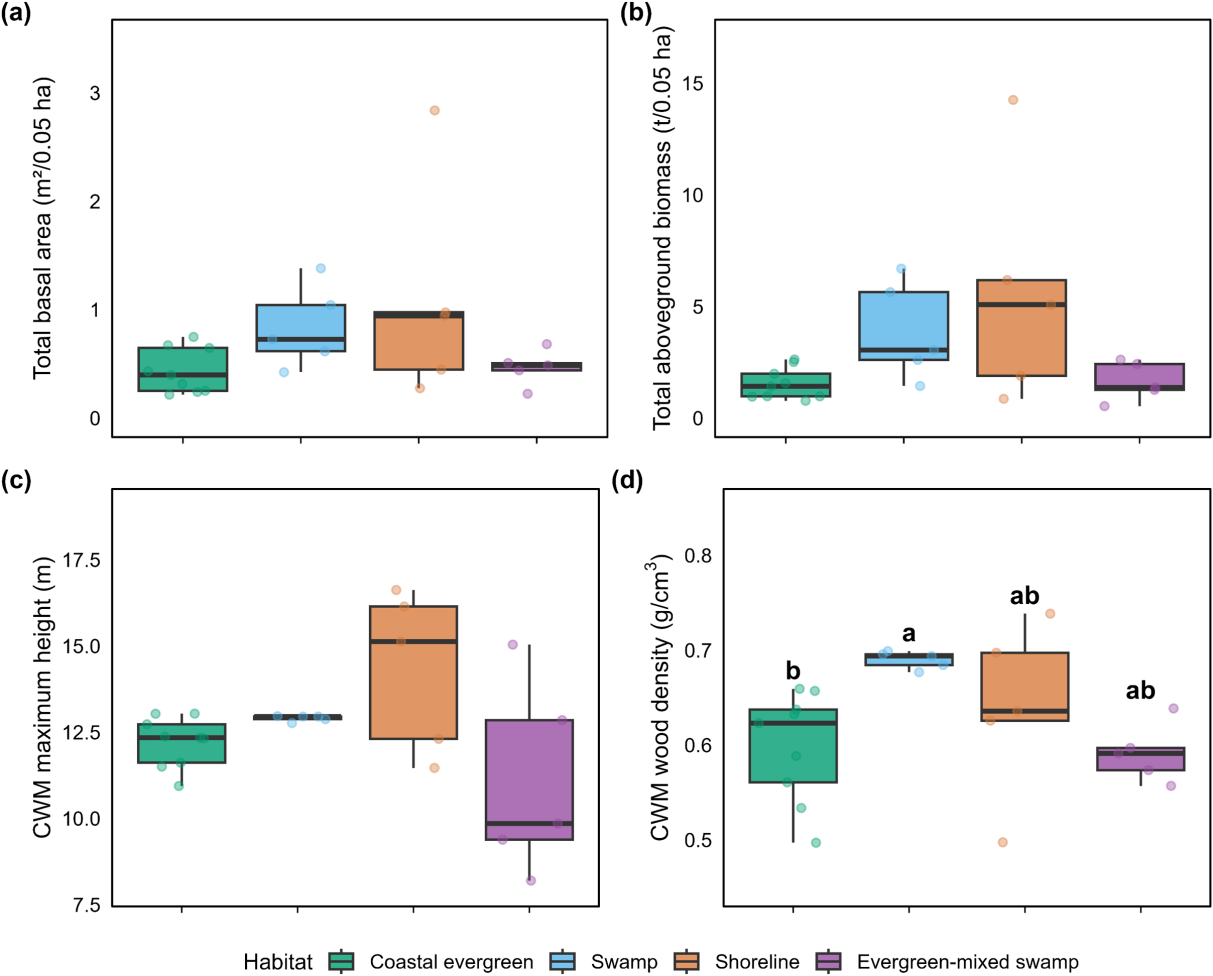
Boxplots comparing structural characteristics and functional traits among habitat types: (a) total basal area; (b) total aboveground biomass; (c) CWM maximum height; (d) CWM wood density. The line within each box represents the median. The box edges represent the first and third quartiles. Whiskers extend to 1.5 times the interquartile range. Points show individual plot values, jittered for clarity. Sample sizes were coastal evergreen (*n* = 9), swamp (*n* = 5), shoreline (*n* = 5), and evergreen–mixed swamp (*n* = 5). Different letters above the boxes indicate statistically significant differences among habitats (Tukey's HSD or Dunn's test, *p* < 0.05, depending on the test used).

Among all structural and functional traits examined, only CWM wood density differed significantly among habitats (*F* (3, 20) = 3.53, *p* = 0.034, η ^2^ = 0.35; Figure 7). The swamp habitat exhibited significantly higher CWM wood density than the coastal evergreen habitat, while no other pairwise differences were detected (Figure 7d).

## 4 Discussion

### 4.1 Habitat Differentiation and Species Diversity Patterns

Variation in tree community composition across the coastal landscape reflects underlying environmental conditions and disturbance regimes. Based on species composition and structural metrics, four distinct habitat types were identified (Figure 3), each showing clear separation in ordination space (Figure 5a). The convex hulls indicated a generally clear separation among habitat groups, suggesting distinct community composition patterns in ordination space. Small areas of overlap were observed in a few cases, most notably between the coastal evergreen community and the shoreline community, where the overlap was limited but clearly visible, indicating partial compositional similarity between these habitats. The first ordination axis (MDS1) was associated with variation in species composition and showed a gradient driven by differences in species dominance, specifically the relative contribution of *M. cajuputi* . This suggests a compositional gradient that may reflect variation in hydrological conditions across habitats, where species composition is partly structured by taxa associated with wetter environments, including *M. cajuputi* . The second axis (MDS2) suggested additional species turnover potentially associated with coastal and disturbance‐related species assemblages, as reflected by the relatively high contribution of the coastal species *Barringtonia asiatica* in the shoreline habitat (Figure 4). The dominance of this species is consistent with the position of the shoreline habitat closest to the coast, where coastal influences are likely stronger than in inland habitats. Patterns of within‐habitat variability were further reflected in multivariate dispersion (Figure 5b). The coastal evergreen, shoreline, and evergreen–mixed swamp habitats exhibited higher dispersion, indicating greater within‐habitat variability in community composition, whereas the swamp habitat was more homogeneous. These differences in dispersion may influence the interpretation of PERMANOVA results, as multivariate differences among groups can be affected by variation in within‐habitat heterogeneity. These patterns may reflect habitat‐specific environmental filtering associated with differences in disturbance intensity and hydrological constraints, which favor certain species while limiting others (Cornwell and Ackerly 2009; Kraft et al. 2008, 2015; Laughlin and Joshi 2015; Siefert 2012; Tameirão et al. 2021). The variation ranges observed in the diversity indices (Figure 6) likely reflect differences in within‐habitat environmental heterogeneity. The coastal evergreen, shoreline, and evergreen–mixed swamp habitats exhibited broader ranges of diversity values, suggesting greater variation among plots in local disturbance intensity and habitat conditions, whereas the swamp habitat showed a narrower range of diversity values, consistent with its lower multivariate dispersion and more homogeneous community composition. Although some overlap was observed among habitat types in the alpha diversity indices, habitats may exhibit similar levels of species richness, diversity, and evenness while differing substantially in species identities and dominance patterns. Therefore, the habitat classification derived from species composition and structural characteristics captured ecological differences that were not fully reflected by alpha diversity metrics alone.

Each habitat corresponds to a distinct environmental and disturbance context. The coastal evergreen habitat is located centrally within the dune landscape and supports high species diversity. Its central location along the shoreline–inland gradient may buffer extreme shoreline disturbance, resulting in lower disturbance intensity that facilitates species coexistence. In contrast, the swamp habitat, located furthest inland, experiences strong hydrological constraints associated with seasonal flooding. The strong dominance of *M. cajuputi* (Figure 4) suggests that hydrological stress may play an important role in limiting species diversity. Because diversity indices were calculated using species proportions based on basal area rather than stem abundance, the disproportionate contribution of *M. cajuputi* to total stand basal area further reduced diversity and evenness values in this habitat. *M. cajuputi* ‐dominated stands represent a distinct habitat type across Southeast Asia and northern Australia (Arifin et al. 2016; Franklin et al. 2007). In peat swamp systems within these regions, stands typically occur in flood‐prone environments on highly acidic soils with acid sulfate conditions associated with pyrite and often form relatively homogeneous stands (Arifin et al. 2016). The evergreen–mixed swamp habitat exhibits transitional characteristics, experiencing moderate disturbance and occasional flooding, allowing swamp species, including *M. cajuputi* , to coexist with species typical of less flood‐prone dune habitats.

Overall, these patterns demonstrate how habitat‐specific environmental conditions and disturbance regimes shape species diversity and community structure. Frequent or intense physical disturbance may influence species coexistence and favor disturbance‐adapted species, whereas differences in disturbance intensity and environmental constraints can contribute to contrasting community structures among habitats (Kim and Ohr 2020; Soliveres and Maestre 2014). Spatial position, together with shoreline–inland disturbance gradients and local habitat characteristics, jointly drives variation in community composition and within‐habitat heterogeneity.

### 4.2 Environmental Variation and Spatial Structuring on Coastal Tree Communities

We hypothesized that recurrent physical disturbance and associated environmental constraints influence coastal tree community composition. Our results are consistent with this hypothesis, as distance from the sea emerged as the variable most strongly associated with community composition and may reflect a shoreline to inland disturbance gradient. In coastal dune systems, proximity to the shoreline is commonly associated with increased exposure to wind, salt spray, and sand burial, which together represent dominant disturbance processes affecting vegetation structure (Du and Hesp 2020; Green and Miller 2019; Hesp 1991; La Bella et al. 2024). Beyond this broad‐scale gradient, spatial position along the east–west axis and elevation were also significantly associated with variation in community composition, indicating that fine‐scale spatial heterogeneity and microtopographic variation further contribute to community differentiation. Although dune morphology was not directly quantified in this study, variation in elevation may partially reflect underlying geomorphological structure and influence local hydrological conditions and disturbance exposure. The absence of detailed dune morphological data represents a limitation of the present study and warrants further investigation.

Together, these findings highlight the combined roles of large‐scale disturbance gradients and fine‐scale spatial heterogeneity in structuring coastal tree communities. The weak but significant correlation between community composition and spatial distance suggests some degree of spatial structuring across the study area. However, community dissimilarity showed a stronger relationship with shoreline distance than with geographic distance among plots, suggesting that the shoreline–inland gradient was more strongly associated with community differentiation than spatial distance alone. Nonetheless, spatial position and microtopographic variation may also contribute to finer‐scale community structure, suggesting that community composition may be influenced by environmental gradients operating at multiple spatial scales.

### 4.3 Structural Metrics and Community Functional Traits

Our results are consistent with the hypothesis that habitats experiencing different environmental constraints and disturbance regimes differ in functional trait composition, particularly wood density. Among all structural and functional traits examined, only community‐weighted mean (CWM) wood density differed significantly across habitats (Figure 7). The absence of significant differences in basal area, aboveground biomass, and CWM maximum height indicating that no significant differences in stand structure were detected among habitats. Therefore, it is possible that ecological differentiation among habitats was expressed more strongly through species composition and, to a lesser extent, functional trait composition, particularly wood density. The large variation observed in aboveground biomass likely reflects the presence of a small number of exceptionally large trees in some plots, a common feature of tropical forest stands. Such individuals can disproportionately influence biomass estimates and contribute to greater variability among plots despite the absence of significant differences among habitats. However, this result should be interpreted cautiously, as the small number of plots per habitat and the high within‐habitat variability in aboveground biomass may have limited statistical power to detect such differences.

The swamp habitat exhibited the highest CWM wood density. This pattern is likely associated with hydrological constraints, as variation in hydrological conditions has been shown to influence wood density in riparian and flood‐prone systems, with species in hydrologically stressful environments often exhibiting more conservative functional strategies (Lawson et al. 2015). The dominance of *M. cajuputi* in this habitat is consistent with this interpretation, as the species has been reported to be well adapted to flooded conditions (Tran et al. 2013). The shoreline habitat, located near the coastal edge, showed CWM wood density values that overlapped with those of the coastal evergreen and swamp habitats. This overlap suggests a community composed of species with varying wood density rather than a single, strongly filtered trait syndrome. Despite variation in median values among habitats, CWM maximum height did not differ significantly. This suggests that tree communities across habitats maintained broadly similar vertical stature despite differences in species composition and environmental conditions. The absence of a clear height response may indicate that the disturbance gradient was insufficient to impose strong filtering on canopy height or that multiple species with contrasting ecological strategies attained comparable maximum heights across habitats.

The coastal evergreen habitat exhibited the lowest CWM wood density, whereas the shoreline and evergreen–mixed swamp habitats showed substantial overlap with both the coastal evergreen and swamp habitats. Lower wood density in the coastal evergreen habitat may indicate reduced investment in structural defense and greater allocation to growth and competitive ability (Chave et al. 2009; Muller‐Landau 2004; Poorter et al. 2008). Together, these patterns suggest that hydrological constraints may play an important role in structuring functional trait composition in the swamp habitat, while the shoreline habitat appears to support a functionally heterogeneous community shaped by recurrent physical disturbance. The coastal evergreen and evergreen–mixed swamp habitats, in turn, appear to support communities with trait compositions distinct from those of the swamp habitat. These findings are consistent with previous studies demonstrating links between hydrological variability and increased wood density in riparian and flood‐prone systems (Lawson et al. 2015), highlighting the importance of conservative functional strategies for tree persistence under challenging environmental conditions.

### 4.4 Influence of Multiple Disturbance Regimes

Coastal tree communities reflect the combined effects of multiple disturbance regimes operating across broad environmental gradients and local‐scale processes. Distance from the sea represents a primary gradient of exposure to recurrent physical disturbance (Green and Miller 2019; La Bella et al. 2024; Munns and Tester 2008). Seasonal disturbances, including monsoon events, further influence plant community composition (Kim et al. 2015), with particularly pronounced effects during periods of strong winds. In this coastal area, the southwest monsoon enhances sediment transport and coastal disturbance, especially in forests near the shoreline (Tharawechrak 2022), reinforcing spatial variation in community structure along the sea–inland gradient (Tharawechrak 2022). In contrast, seasonal flooding represents a locally driven hydrological disturbance regulated by water table dynamics, rainfall, and dune geomorphology rather than shoreline distance (Dwyer et al. 2021). Flooding may act as an environmental filter favoring species with flood‐tolerant traits, such as higher wood density (Lawson et al. 2015). Its effects are strongly mediated by dune topography, with low‐lying dune slacks experiencing prolonged inundation relative to higher dune ridges (Schat and van Beckhoven 1991). Because flooding regimes, groundwater dynamics, and soil conditions were not quantified directly in this study, their influences are inferred from established ecological relationships and observed patterns along the shoreline–inland gradient.

Beyond these recurrent disturbance processes, the study area bears the legacy of a rare, high‐intensity pulse disturbance in the form of a past tsunami in 2004 that affected parts of the coastal evergreen and shoreline habitats. Although spatially limited, this event likely caused short‐term tree mortality and may have contributed to longer‐term changes in soil salinity and sediment deposition. The persistence of surviving individuals suggests either selection for tolerant species or local microhabitat buffering, whereby surrounding shoreline forests may have reduced wave energy, salinity intrusion, or sediment disturbance during the event (Sridith 2006). Such infrequent but extreme disturbances can interact with ongoing gradient‐driven and hydrological processes to generate additional spatial heterogeneity that may contribute to observed patterns of species distribution and community structure. Together, the interaction of broad‐scale disturbance gradients, local flooding regimes, and episodic high‐intensity events creates complex selective pressures that, in combination with spatial and topographic variation, structure coastal tree communities.

### 4.5 Implications for Coastal Habitat Management

Our study demonstrates that coastal dune tree communities are structured by multiple interacting disturbance regimes operating across spatial scales. These recurrent physical and hydrological disturbances may contribute to ecological differentiation among habitats and influence species persistence through their functional traits, shaping community structure and ecosystem functioning. A trait‐based perspective therefore provides a useful framework for predicting ecosystem resilience and guiding adaptive management in complex coastal landscapes.

In the swamp habitat, community structure is strongly shaped by recurrent hydrological disturbance associated with rainfall and water table dynamics, favoring species with conservative functional strategies, particularly *M. cajuputi* (Figure 4). The dominance of *M. cajuputi* reflects adaptation to repeated flooding and waterlogging (Tran et al. 2013) and has important implications for ecosystem carbon storage. Because this species contributes substantially to carbon sequestration (Dang et al. 2023; Tran et al. 2015; Walker and Zinnert 2022), its high basal area (Table A1) suggests a potentially important role in regulating carbon dynamics within the system. The persistence of *Melaleuca* species under past climatic variability further indicates tolerance to hydrological stress (Tran et al. 2013). Under projected increases in monsoon intensity and flooding frequency in Southeast Asia (Hariadi et al. 2024; IPCC 2022), species with high wood density and flood tolerance may gain a competitive advantage, potentially influencing future carbon storage trajectories (Tran et al. 2015). Incorporating functional trait information into coastal management and restoration planning may therefore enhance ecosystem stability in flood‐prone habitats. However, the ecological role of *M. cajuputi* remains context dependent, as low‐lying deltaic wetlands may be particularly vulnerable to sea‐level rise (Dang et al. 2023), potentially limiting its long‐term persistence under future coastal change.

At the landscape scale, the clear ecological differentiation among shoreline, inland swamp, and transitional habitats highlights the importance of spatially explicit management in coastal dune systems. Conservation strategies that recognize variation in disturbance regimes, hydrological conditions, and functional trait composition may help maintain biodiversity, structural complexity, and carbon storage across the coastal mosaic. In protected coastal regions such as the Andaman coast of Thailand, incorporating habitat‐specific ecological information into zoning and management planning could enhance long‐term ecosystem resilience under increasing climatic and anthropogenic pressures. These findings provide baseline ecological evidence that may inform future coastal landscape management.

## 5 Conclusions

This study suggests that coastal dune plant communities are structured by interacting disturbance regimes operating across spatial gradients, particularly those associated with shoreline proximity and potential hydrological influences. While distance from the sea was associated with variation in species composition, functional trait variation was primarily linked to inferred hydrological disturbance. Differences in community‐weighted mean wood density across habitats suggest potential trait‐based adaptation to flooding stress. Our findings highlight the importance of considering both disturbance gradients and functional trait composition when evaluating coastal ecosystem resilience. Because disturbance was not measured directly and shoreline distance was used as a proxy for multiple disturbance processes, the observed relationships should be interpreted as pattern‐based ecological inference rather than strong causal evidence. Future research integrating additional functional traits and environmental variables may further clarify the mechanisms underlying community differentiation in complex coastal landscapes. These findings provide baseline ecological evidence that may inform future spatial planning within coastal protected area networks.

## Author Contributions

**Kanokporn Kaewsong:** conceptualization (lead), data curation (lead), formal analysis (lead), funding acquisition (lead), investigation (lead), methodology (equal), project administration (equal), resources (lead), software (lead), validation (equal), visualization (lead), writing – original draft (lead), writing – review and editing (equal). **Ekaphan Kraichak:** conceptualization (equal), methodology (equal), supervision (equal), visualization (equal), writing – review and editing (equal). **Chia‐Hao Chang‐Yang:** conceptualization (equal), methodology (equal), supervision (equal), writing – review and editing (equal). **Atiwat Boonrit:** conceptualization (supporting), data curation (supporting), investigation (supporting), project administration (supporting), writing – review and editing (equal). **Prarop Plangngan:** project administration (supporting), resources (supporting), writing – review and editing (equal). **Sangsan Phumsathan:** conceptualization (equal), funding acquisition (supporting), supervision (lead), writing – review and editing (equal).

## Funding

This research was funded by Kasetsart University Research and Development Institute, KURDI (Project No. YF(KU)5.68).

## Conflicts of Interest

The authors declare no conflicts of interest.

## Supporting information


**Table A1** Relative contribution of tree species to basal area in Khao Lampi–Hat Thai Mueang National Park. Species are ranked in descending order of basal area contribution.

**Table S1:** Plot‐level structural and functional attributes of the 24 study plots.
**Table S2:** Tree species recorded in the 24 study plots, including species and family.
**Figure S1:** Distance‐based redundancy analysis (db‐RDA) ordination of tree community composition in relation to distance from the sea, elevation, and spatial coordinates. Arrows represent the explanatory variables included in the model.

## Data Availability

All data supporting the findings of this study are available as Supporting Information.
