# Supplementary material for: Habitat Differentiation and Trait Variation Across Disturbance Gradients in Coastal Plant Communities of the Andaman Coast, Thailand
Source: Ecol Evol. 2026 Jul 3;16(7):e73964. doi: 10.1002/ece3.73964 (PMC13331752; doi:10.1002/ece3.73964)
Supplement: Supplementary file 1 — Table A1 Relative contribution of tree species to basal area in Khao Lampi–Hat Thai Mueang National Park. Species are ranked in descending order of basal area contribution. [file ECE3-16-e73964-s002.docx]

APPENDIX A

**Table A1.** Relative contribution of tree species to basal area in Khao Lampi–Hat Thai Mueang National Park. Species are ranked in descending order of basal area contribution.

| **Rank** | **Species** | **Basal area (%)** | **Cumulative (%)** |
| --- | --- | --- | --- |
| 1 | *Melaleuca cajuputi* | 27.62 | 27.62 |
| 2 | *Barringtonia asiatica* | 10.56 | 38.18 |
| 3 | *Vitex canescens* | 6.56 | 44.74 |
| 4 | *Heritiera littoralis* | 6.37 | 51.11 |
| 5 | *Diospyros malabarica* | 5.94 | 57.05 |
| 6 | *Shorea roxburghii* | 5.63 | 62.68 |
| 7 | *Acacia mangium* | 4.75 | 67.43 |
| 8 | *Terminalia catappa* | 3.71 | 71.14 |
| 9 | *Casuarina equisetifolia* | 2.83 | 73.97 |
| 10 | *Ficus caulocarpa* | 2.51 | 76.48 |
| 11 | *Acronychia pedunculata* | 2.45 | 78.93 |
| 12 | *Glycosmis pentaphylla* | 2.22 | 81.15 |
| 13 | *Lepisanthes rubiginosa* | 2.11 | 83.26 |
| 14 | *Syzygium antisepticum* | 2.02 | 85.28 |
| 15 | *Atalantia monophylla* | 1.50 | 86.78 |
| 16 | *Ficus consociata* | 1.43 | 88.21 |
| 17 | *Calophyllum inophyllum* | 1.29 | 89.50 |
| 18 | *Syzygium grande* | 1.28 | 90.78 |
| 19 | *Pandanus odorifer* | 1.10 | 91.88 |
| 20 | *Mischocarpus sundaicus* | 1.07 | 92.95 |
| 21 | *Diospyros vera* | 0.92 | 93.87 |
| 22 | *Ficus retusa* | 0.78 | 94.65 |
| 23 | *Guettarda speciosa* | 0.73 | 95.38 |
| 24 | *Syzygium cumini* | 0.69 | 96.07 |
| 25 | *Melicope lunu-ankenda* | 0.67 | 96.74 |
| 26 | *Cerbera odollam* | 0.52 | 97.26 |
| 27 | *Gomphia serrata* | 0.48 | 97.74 |
| 28 | *Ficus* sp. | 0.40 | 98.14 |
| 29 | *Syzygium siamense* | 0.29 | 98.43 |
| 30 | *Diospyros pilosanthera* | 0.22 | 98.65 |
| 31 | *Planchonella obovata* | 0.20 | 98.85 |
| 32 | *Ardisia elliptica* | 0.20 | 99.05 |
| 33 | *Buchanania arborescens* | 0.19 | 99.24 |
| 34 | *Ochrosia oppositifolia* | 0.13 | 99.37 |
| 35 | *Syzygium claviflorum* | 0.09 | 99.46 |
| 36 | *Catunaregam spathulifolia* | 0.09 | 99.55 |
| 37 | *Hibiscus tiliaceus* | 0.09 | 99.64 |
| 38 | *Melientha suavis* | 0.06 | 99.70 |
| 39 | *Claoxylon indicum* | 0.05 | 99.75 |
| 40 | *Memecylon plebejum* | 0.04 | 99.79 |
| 41 | *Cinnamomum iners* | 0.04 | 99.83 |
| 42 | *Premna serratifolia* | 0.03 | 99.86 |
| 43 | *Elaeocarpus rugosus* | 0.03 | 99.89 |
| 44 | *Calophyllum pisiferum* | 0.03 | 99.92 |
| 45 | *Garcinia vilersiana* | 0.02 | 99.94 |
| 46 | *Memecylon lilacinum* | 0.02 | 99.96 |
| 47 | *Memecylon caeruleum* | 0.02 | 99.98 |
| 48 | *Anacardium occidentale* | 0.01 | 99.99 |
| 49 | *Vatica odorata* | 0.01 | 100.00 |
