# Supplementary material for: Habitat Differentiation and Trait Variation Across Disturbance Gradients in Coastal Plant Communities of the Andaman Coast, Thailand
Source: Ecol Evol. 2026 Jul 3;16(7):e73964. doi: 10.1002/ece3.73964 (PMC13331752; doi:10.1002/ece3.73964)
Supplement: Supplementary file 2 — Table S1: Plot‐level structural and functional attributes of the 24 study plots. Table S2: Tree species recorded in the 24 study plots, including species and family. Figure S1: Distance‐based redundancy analysis (db‐RDA) ordination of tree community composition in relation to distance from the sea, elevation, and spatial coordinates. Arrows represent the explanatory variables included in the model. [file ECE3-16-e73964-s001.docx]

Table S1 Plot-level structural and functional attributes of the 24 study plots.

| Plot10x50 | Basal area (m²) | Aboveground biomass (t/0.05 ha) | CWM maximum height (m) | CWM wood density (g/cm³) | Individual tree | Habitat |
| --- | --- | --- | --- | --- | --- | --- |
| 01 | 0.942 | 5.084634 | 15.1 | 0.738 | 57 | 3 |
| 02 | 0.446 | 1.895006 | 12.3 | 0.625 | 48 | 3 |
| 03 | 0.217 | 0.777918 | 10.9 | 0.659 | 29 | 1 |
| 04 | 0.439 | 2.619472 | 15.0 | 0.638 | 28 | 4 |
| 05 | 0.224 | 0.536134 | 9.3 | 0.557 | 44 | 4 |
| 06 | 0.507 | 1.268824 | 8.1 | 0.573 | 90 | 4 |
| 07 | 0.976 | 6.181776 | 16.1 | 0.697 | 43 | 3 |
| 08 | 0.242 | 0.975783 | 13.0 | 0.637 | 32 | 1 |
| 09 | 0.647 | 2.514478 | 11.5 | 0.632 | 67 | 1 |
| 10 | 0.673 | 1.991412 | 11.6 | 0.560 | 57 | 1 |
| 11 | 0.398 | 1.565227 | 12.3 | 0.657 | 47 | 1 |
| 12 | 0.486 | 1.370549 | 9.8 | 0.597 | 66 | 4 |
| 13 | 0.276 | 0.865207 | 11.4 | 0.635 | 54 | 3 |
| 14 | 0.749 | 2.627938 | 12.3 | 0.497 | 33 | 1 |
| 15 | 0.430 | 1.423748 | 13.0 | 0.623 | 38 | 1 |
| 16 | 0.617 | 2.610494 | 12.9 | 0.699 | 59 | 2 |
| 17 | 1.043 | 5.645403 | 12.9 | 0.695 | 37 | 2 |
| 18 | 0.425 | 1.441535 | 12.7 | 0.693 | 32 | 2 |
| 19 | 2.836 | 14.25623 | 16.6 | 0.497 | 58 | 3 |
| 20 | 0.251 | 0.981133 | 12.7 | 0.588 | 42 | 1 |
| 21 | 0.314 | 0.961048 | 12.3 | 0.533 | 43 | 1 |
| 22 | 0.681 | 2.427395 | 12.84997 | 0.591 | 61 | 4 |
| 23 | 1.381 | 6.698739 | 12.96398 | 0.684 | 110 | 2 |
| 24 | 0.727 | 3.050651 | 12.87905 | 0.677 | 82 | 2 |

**Notes:** CWM maximum height = community-weighted mean maximum height; CWM wood density = community-weighted mean wood density. Each plot measured 10 × 50 m (0.05 ha). Habitat codes are defined as follows: 1 = Coastal evergreen, 2 = Swamp, 3 = Shoreline, and 4 = Evergreen–mixed swamp.

Table S2 Tree species recorded in the 24 study plots, including species and family.

|  | Family | Species |
| --- | --- | --- |
| 1 | Anacardiaceae | *Anacardium occidentale* |
| 2 | Anacardiaceae | *Buchanania arborescens* |
| 3 | Apocynaceae | *Cerbera odollam* |
| 4 | Apocynaceae | *Ochrosia oppositifolia* |
| 5 | Calophyllaceae | *Calophyllum inophyllum* |
| 6 | Calophyllaceae | *Calophyllum pisiferum* |
| 7 | Casuarinaceae | *Casuarina equisetifolia* |
| 8 | Clusiaceae | *Garcinia vilersiana* |
| 9 | Combretaceae | *Terminalia catappa* |
| 10 | Dipterocarpaceae | *Shorea roxburghii* |
| 11 | Dipterocarpaceae | *Vatica odorata* |
| 12 | Ebenaceae | *Diospyros malabarica* |
| 13 | Ebenaceae | *Diospyros pilosanthera* |
| 14 | Ebenaceae | *Diospyros vera* |
| 15 | Elaeocarpaceae | *Elaeocarpus rugosus* |
| 16 | Euphorbiaceae | *Claoxylon indicum* |
| 17 | Fabaceae | *Acacia mangium* |
| 18 | Lamiaceae | *Premna serratifolia* |
| 19 | Lamiaceae | *Vitex canescens* |
| 20 | Lauraceae | *Cinnamomum iners* |
| 21 | Lecythidaceae | *Barringtonia asiatica* |
| 22 | Malvaceae | *Heritiera littoralis* |
| 23 | Malvaceae | *Hibiscus tiliaceus* |
| 24 | Melastomaceae | *Memecylon caeruleum* |
| 25 | Melastomaceae | *Memecylon lilacinum* |
| 26 | Melastomaceae | *Memecylon plebejum* |
| 27 | Moraceae | *Ficus caulocarpa* |
| 28 | Moraceae | *Ficus consociata* |
| 29 | Moraceae | *Ficus retusa* |
| 30 | Moraceae | *Ficus* sp. |
| 31 | Myrtaceae | *Melaleuca cajuputi* |
| 32 | Myrtaceae | *Syzygium antisepticum* |
| 33 | Myrtaceae | *Syzygium claviflorum* |
| 34 | Myrtaceae | *Syzygium cumini* |
| 35 | Myrtaceae | *Syzygium grande* |
| 36 | Myrtaceae | *Syzygium siamense* |
| 37 | Ochnaceae | *Gomphia serrata* |
| 38 | Opiliaceae | *Melientha suavis* |
| 39 | Pandanaceae | *Pandanus odorifer* |
| 40 | Primulaceae | *Ardisia elliptica* |
| 41 | Rubiaceae | *Catunaregam spathulifolia* |
| 42 | Rubiaceae | *Guettarda speciosa* |
| 43 | Rutaceae | *Acronychia pedunculata* |
| 44 | Rutaceae | *Atalantia monophylla* |
| 45 | Rutaceae | *Glycosmis pentaphylla* |
| 46 | Rutaceae | *Melicope lunu-ankenda* |
| 47 | Sapindaceae | *Lepisanthes rubiginosa* |
| 48 | Sapindaceae | *Mischocarpus sundaicus* |
| 49 | Sapotaceae | *Planchonella obovata* |


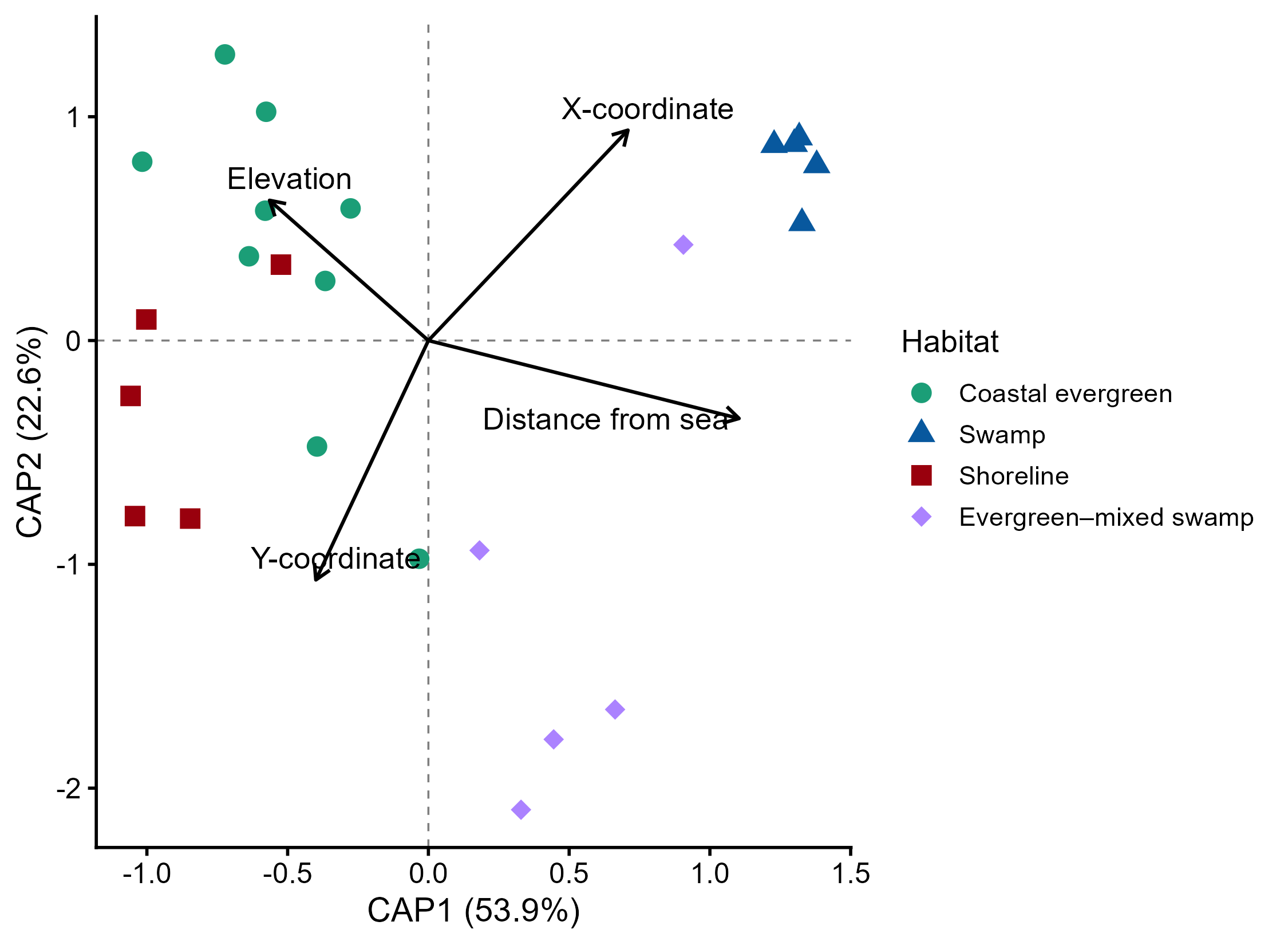


FIGURE S1 Distance-based redundancy analysis (db-RDA) ordination of tree community composition in relation to distance from the sea, elevation, and spatial coordinates. Arrows represent the explanatory variables included in the model.
